# From Pancytopenia to Diagnosis: Visceral Leishmaniasis Identified Through Primary Care Assessment

**DOI:** 10.7759/cureus.105066

**Published:** 2026-03-11

**Authors:** Francisco Oliveira, Virginia Martínez, Carla Moreira, Isabelle Carrilho, Teresa Pipa, Luís Neves, Daniela Azevedo, Ana Teresa Alves, Beatriz Garcia, José Rei

**Affiliations:** 1 Family Medicine, Unidade de Saúde Familiar (USF) Lusitana, Unidade Local de Saúde Viseu Dão-Lafões, Viseu, PRT; 2 Pathology and Laboratory Medicine, Unidade Local de Saúde Viseu Dão-Lafões, Viseu, PRT; 3 Family and Community Medicine, Unidade de Saúde Familiar (USF) Lusitana, Unidade Local de Saúde Viseu Dão-Lafões, Viseu, PRT

**Keywords:** family medicine, liposomal amphotericin b, pancytopenia, primary medical care, visceral leishmaniasis (vl)

## Abstract

Visceral leishmaniasis is a potentially life-threatening systemic parasitic infection caused by protozoa of the genus Leishmania and transmitted by sandflies. Although uncommon in Portugal, it should be considered in the differential diagnosis of prolonged fever associated with pancytopenia and hepatosplenomegaly, even in immunocompetent individuals. We report a case of a previously healthy young adult who initially presented to an urgent Family Medicine consultation in primary care, where pancytopenia was identified and prompted timely referral to secondary care. Diagnosis was confirmed by the detection of Leishmania amastigotes in bone marrow aspirate during haematological investigation. Treatment with liposomal amphotericin B resulted in complete clinical and laboratory recovery. This case highlights the importance of careful primary care assessment, recognition of red flags, and effective coordination between primary and secondary care in non-endemic settings.

## Introduction

Visceral leishmaniasis (VL), also known as kala-azar, is a severe systemic parasitic disease caused by intracellular protozoa of the genus Leishmania and transmitted through the bite of infected sandflies. Although VL is endemic in several tropical and subtropical regions worldwide, it remains relatively rare in Portugal, where Leishmania infantum is the most frequently identified species and dogs act as the primary reservoir [1-3].

Clinical presentation is often non-specific and includes prolonged fever, asthenia, weight loss, hepatosplenomegaly, and cytopenias. These features frequently lead to diagnostic delay and initial suspicion of haematological malignancy, autoimmune disease, or other systemic infections. Definitive diagnosis relies on identification of the parasite in tissue samples, most commonly bone marrow. The treatment of choice is liposomal amphotericin B. From a primary care perspective, early recognition of red flags and urgent referral are essential to achieve favourable outcomes [3-5].

## Case presentation

 A 32-year-old man, previously healthy and living in a rural area, presented to an urgent Family Medicine consultation with a one-month history of marked asthenia, myalgia, dizziness triggered by head movements, evening fever (up to 38°C), night sweats, and unintentional weight loss of approximately 10 kg. He denied respiratory or gastrointestinal symptoms.

Targeted history revealed no domestic animals at home, occasional visits to a small farm with poultry, and sporadic consumption of untreated spring water. There were no recent travels, sick contacts, or other relevant epidemiological exposures.

Physical examination revealed palpable splenomegaly approximately 3-4 cm below the left costal margin, with no other relevant findings. Urgent laboratory tests requested in primary care showed pancytopenia (Table 1). Given the combination of prolonged fever, constitutional symptoms, splenomegaly, and pancytopenia, the family physician contacted the on-call haematology service, and the patient was assessed in hospital four days later.

**Table 1:** Laboratory findings and diagnostic investigations. CRP: C-reactive protein, ESR: erythrocyte sedimentation rate, CK: creatine kinase, HDL: high-density lipoprotein, LDH: lactate dehydrogenase, ADA: adenosine deaminase, STI: sexually transmitted infection

Date	Laboratory parameter	Result	Reference range
07/04	Leukocytes (×10⁹/L)	2.9	4.0–11.0
	Haemoglobin (g/dL)	12.2	12–16
	Platelets (×10⁹/L)	128	150–400
	CRP (mg/L)	41	<5
	Triglycerides (mg/dL)	262	<150
	Total cholesterol (mg/dL)	75	<200
	HDL cholesterol (mg/dL)	13	>40
	CK (IU/L)	55	30–200
14/04	Leukocytes (×10⁹/L)	2.9	4.0–11.0
	Haemoglobin (g/dL)	11.9	12–16
	Platelets (×10⁹/L)	87	150–400
	CRP (mg/L)	32.7	<5
	ESR (mm/h)	15	<20
	LDH (IU/L)	289	140–280
	ADA (IU/L)	71	<40
	IgG (mg/dL)	3313	700–1600
	Ferritin (ng/mL)	1640	30–400
	Autoimmune profile	Negative	—
	STI serologies	Negative	—
	Peripheral blood smear (Wright–Giemsa stain, ×1000 magnification)	Lymphocytic pleomorphism suggestive of lymphocytic activation. No platelet aggregates were observed.	
17/04	Bone marrow aspirate	Leishmania amastigotes identified	—
28/04	Leukocytes (×10⁹/L)	4.48	4.0–11.0
	Haemoglobin (g/dL)	11.3	12–16
	Platelets (×10⁹/L)	162	150–400
	CRP (mg/L)	13.2	<5
	Leishmania serology (IgG)	Positive	—
	Leishmania serology (IgM)	Negative	—
15/07	Leukocytes (×10⁹/L)	5.0	4.0–11.0
	Haemoglobin (g/dL)	15.8	12–16
	Platelets (×10⁹/L)	197	150–400
	CRP (mg/L)	0.2	<5

**Figure 1:**
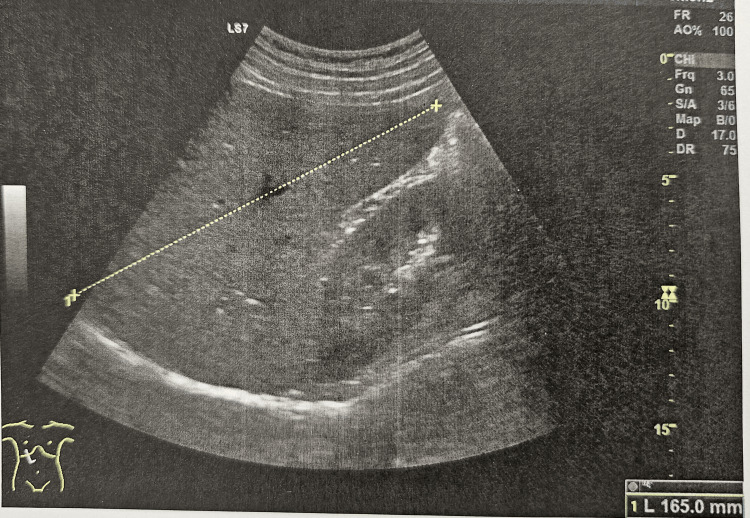
Ultrasound image of hepatomegaly.

**Figure 2:**
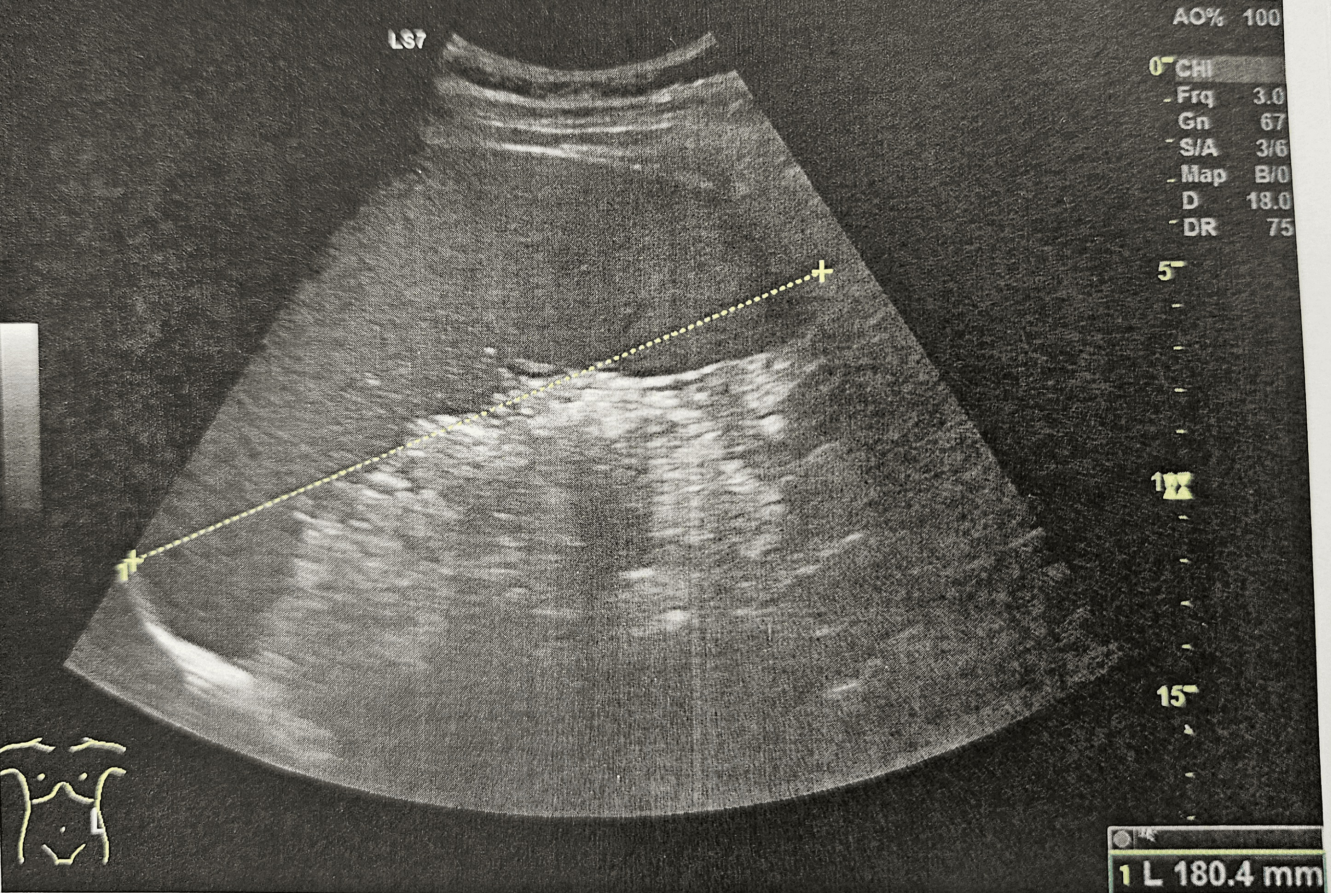
Ultrasound image of splenomegaly.

**Figure 3:**
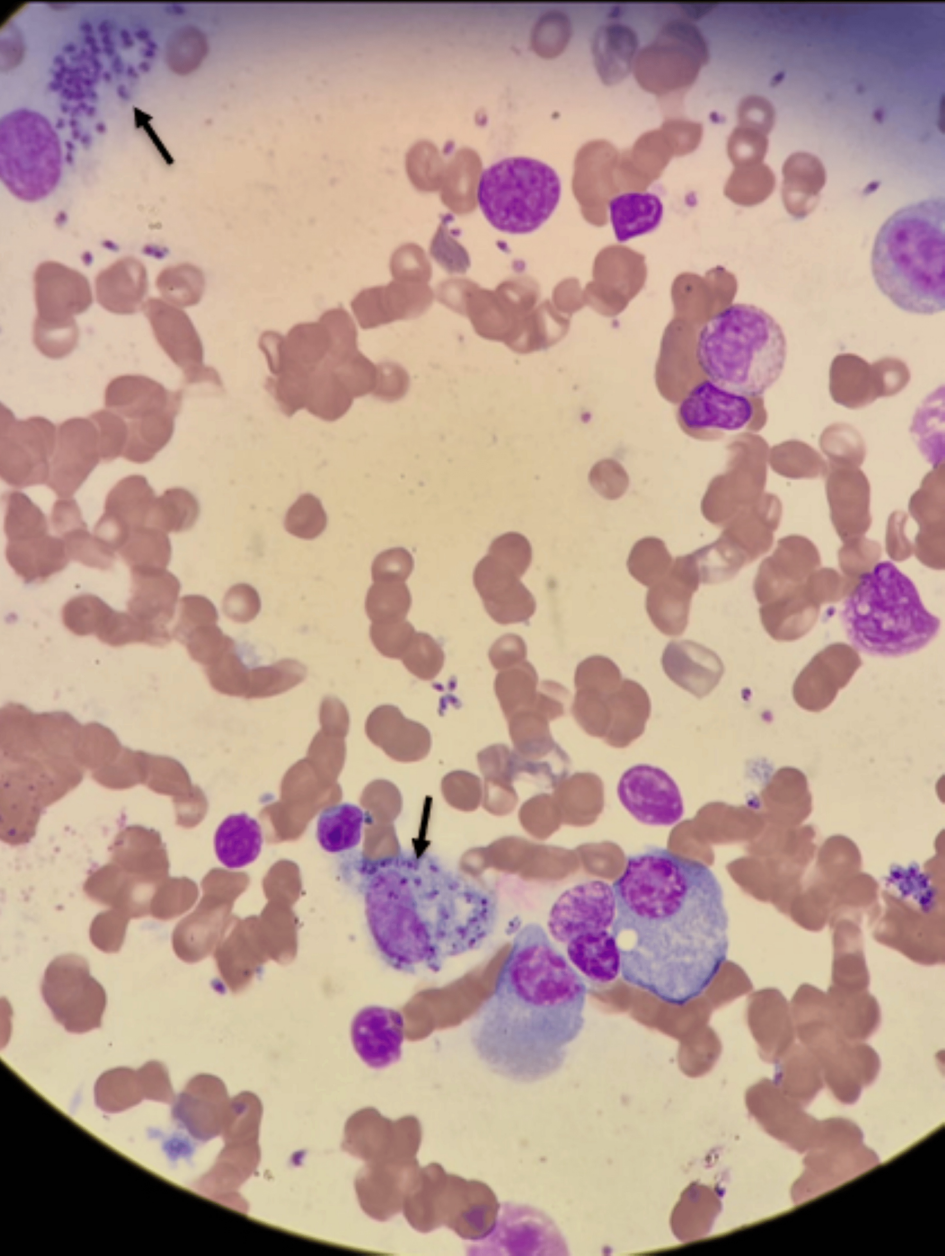
Bone marrow aspirate showing intracellular Leishmania amastigotes. The bone marrow aspirate smear revealed macrophages containing numerous intracellular and extracellular Leishmania amastigotes (May–Grünwald–Giemsa stain, ×1000).

**Figure 4:**
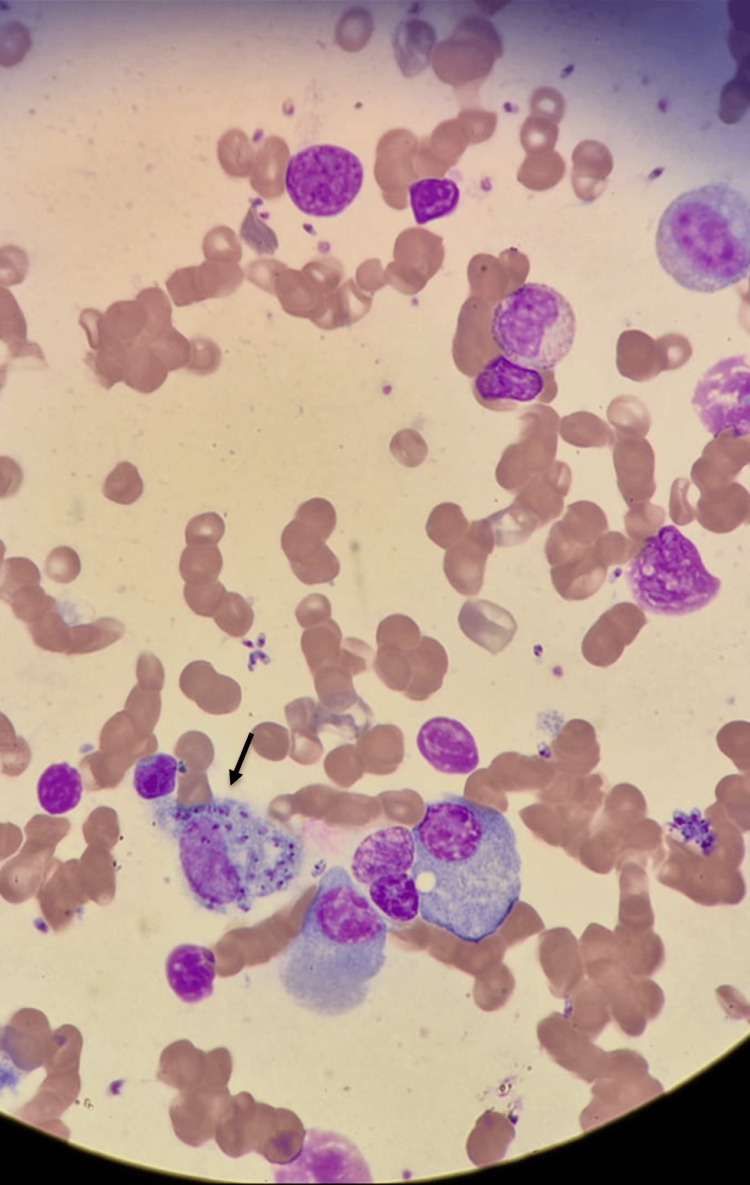
Another view highlighting Leishmania amastigotes within macrophages. Another view with macrophages containing numerous intracellular and extracellular Leishmania amastigotes (May–Grünwald–Giemsa stain, ×1000).

In hospital, pancytopenia was confirmed and further investigation initiated. Abdominal ultrasound demonstrated mild hepatomegaly (16.5 cm, Figure 1) and homogeneous splenomegaly (18 cm, Figure 2). Bone marrow aspirate revealed hypocellularity with the presence of Leishmania amastigotes, identified by the clinical pathology laboratory, confirming the diagnosis of visceral leishmaniasis (Figures 3, 4). Immunophenotyping and karyotype (46,XY) showed no pathological abnormalities.

The patient was admitted under Infectious Diseases and treated with liposomal amphotericin B (3 mg/kg/day for seven days) without complications. Progressive clinical improvement and normalisation of haematological and inflammatory parameters were observed, and the patient was discharged on day seven, with follow-up in Infectious Diseases and Family Medicine.

## Discussion

Although visceral leishmaniasis is rare in Portugal, it should be considered in the differential diagnosis of prolonged fever associated with pancytopenia and splenomegaly, even in immunocompetent patients without clear epidemiological risk factors. Absence of domestic animals does not exclude the diagnosis, as exposure may be indirect or environmental [1,3-5].

Infectious diseases are important causes of bone marrow suppression and pancytopenia and should be considered in the differential diagnosis of patients presenting with unexplained cytopenias. Viral infections such as human immunodeficiency virus, Epstein-Barr virus, hepatitis viruses, cytomegalovirus, parvovirus B19, and dengue virus may impair hematopoiesis. Bacterial infections, including enteric fever and severe sepsis, may also cause transient marrow suppression. Parasitic diseases, particularly visceral leishmaniasis and malaria, are well-recognized causes of pancytopenia in endemic regions due to bone marrow involvement and hypersplenism [6,7].

The non-specific presentation often leads to diagnostic delay and initial suspicion of haematological malignancy. In this context, basic laboratory testing in primary care can uncover potentially serious underlying conditions and support early referral. Bone marrow examination plays a crucial role not only in excluding malignancy but also in identifying infectious causes [2-5].

This report does not aim to highlight a rare diagnosis per se, but rather the clinical reasoning and decision-making process in primary care when faced with prolonged fever and pancytopenia. It underscores the importance of vigilance in primary care and effective communication between levels of care to ensure timely diagnosis, treatment, and continuity of follow-up [3-5].

## Conclusions

Visceral leishmaniasis should be included in the differential diagnosis of pancytopenia and prolonged fever associated with splenomegaly, even in low-endemic regions and in the absence of clear epidemiological risk factors. Early recognition of red flags in primary care, combined with effective coordination between primary and secondary healthcare services, is essential for timely diagnosis and favourable clinical outcomes.
